# Feasibility of a novel non-invasive swab technique for serial whole-exome sequencing of cervical tumors during chemoradiation therapy

**DOI:** 10.1371/journal.pone.0274457

**Published:** 2022-10-06

**Authors:** Julianna K. Bronk, Chiraag Kapadia, Xiaogang Wu, Bhavana V. Chapman, Rui Wang, Tatiana V. Karpinets, Xingzhi Song, Andrew M. Futreal, Jianhua Zhang, Ann H. Klopp, Lauren E. Colbert

**Affiliations:** 1 Department of Radiation Oncology, The University of Texas MD Anderson Cancer Center, Houston, Texas, United States of America; 2 Department of Molecular and Cellular Biology, Baylor College of Medicine, Houston, Texas, United States of America; 3 Department of Genomic Medicine, The University of Texas MD Anderson Cancer Center, Houston, Texas, United States of America; Flinders University, AUSTRALIA

## Abstract

**Background:**

Clinically relevant genetic predictors of radiation response for cervical cancer are understudied due to the morbidity of repeat invasive biopsies required to obtain genetic material. Thus, we aimed to demonstrate the feasibility of a novel noninvasive cervical swab technique to (1) collect tumor DNA with adequate throughput to (2) perform whole-exome sequencing (WES) at serial time points over the course of chemoradiation therapy (CRT).

**Methods:**

Cervical cancer tumor samples from patients undergoing chemoradiation were collected at baseline, at week 1, week 3, and at the completion of CRT (week 5) using a noninvasive swab-based biopsy technique. Swab samples were analyzed with whole-exome sequencing (WES) with mutation calling using a custom pipeline optimized for shallow whole-exome sequencing with low tumor purity (TP). Tumor mutation changes over the course of treatment were profiled.

**Results:**

216 samples were collected and successfully sequenced for 70 patients (94% of total number of tumor samples collected). A total of 33 patients had a complete set of samples at all four time points. The mean mapping rate was 98% for all samples, and the mean target coverage was 180. Estimated TP was greater than 5% for all samples. Overall mutation frequency decreased during CRT but mapping rate and mean target coverage remained at >98% and >180 reads at week 5.

**Conclusion:**

This study demonstrates the feasibility and application of a noninvasive swab-based technique for WES analysis which may be applied to investigate dynamic tumor mutational changes during treatment to identify novel genes which confer radiation resistance.

## Introduction

The global burden of cervical cancer is growing despite the development of HPV vaccines aimed at disease prevention [1]. In 2018, approximately 570,000 new cases of cervical cancer were diagnosed worldwide, resulting in more than 311,000 deaths [2]. High-risk human papillomaviruses (HPVs) are essential in cervical dysplasia and carcinogenesis and cause most cervical cancers [3]. Multimodality therapy is the standard of care for treating locally advanced disease and involves daily external beam radiation treatment, brachytherapy, and weekly chemotherapy [4]. The rate of tumor regression during chemoradiotherapy (CRT) is variable and is strongly associated with survival [5, 6]; however, predictive markers of radiation treatment sensitivity and resistance are currently unknown.

Whole-exome sequencing (WES) technology is a powerful tool that allows for comprehensive analysis of the frequency of somatic mutations, overall tumor mutational burden, and single nucleotide human exome variants (SNVs), which can lead to the identification of pathways that may be functionally significant in cancer outcomes [7].

Previous comprehensive studies of cervical cancer genomics have relied on the analysis of untreated tumors, and none have identified predictors of radiation response [8, 9]. This is mainly due to the challenges of repeated biopsy sampling over the course of the therapy, which are logistically challenging to acquire and entail significant patient morbidity. Nevertheless, cervical tumors are an ideal setting for the serial study of treatment response because tumors can be readily monitored by physical exam and are accessible for sampling through the course of chemoradiotherapy. Encouragingly, successful non-invasive swab-based sample acquisition of DNA has been demonstrated in the screening and diagnosis of precancerous and cancerous lesions in the oral cavity and detection of HPV-associated cutaneous lesions by PCR [10–13].

In this feasibility study, we hypothesize that swab-based sampling of cervical tumors can serve as a robust noninvasive method to acquire tumor specimens for WES. Using swab-based biopsies collected prospectively in 70 patients undergoing CRT for newly diagnosed cervical cancer, the objectives of this study are to: (1) acquire tumor DNA samples adequate for WES in the majority of patients and (2) develop a custom pipeline optimized for shallow WES with low tumor purity (TP) to facilitate mutation calling. Feasibility of this novel non-invasive approach will be essential for a planned follow up study aiming to link mutational landscape changes in the context of disease response in an effort to establish biomarker predictors for treatment response and identify potential drivers of treatment resistance in patients with cervical cancer undergoing definitive CRT.

## Materials and methods

### Patient population and treatment characteristics

**Fig 1:**
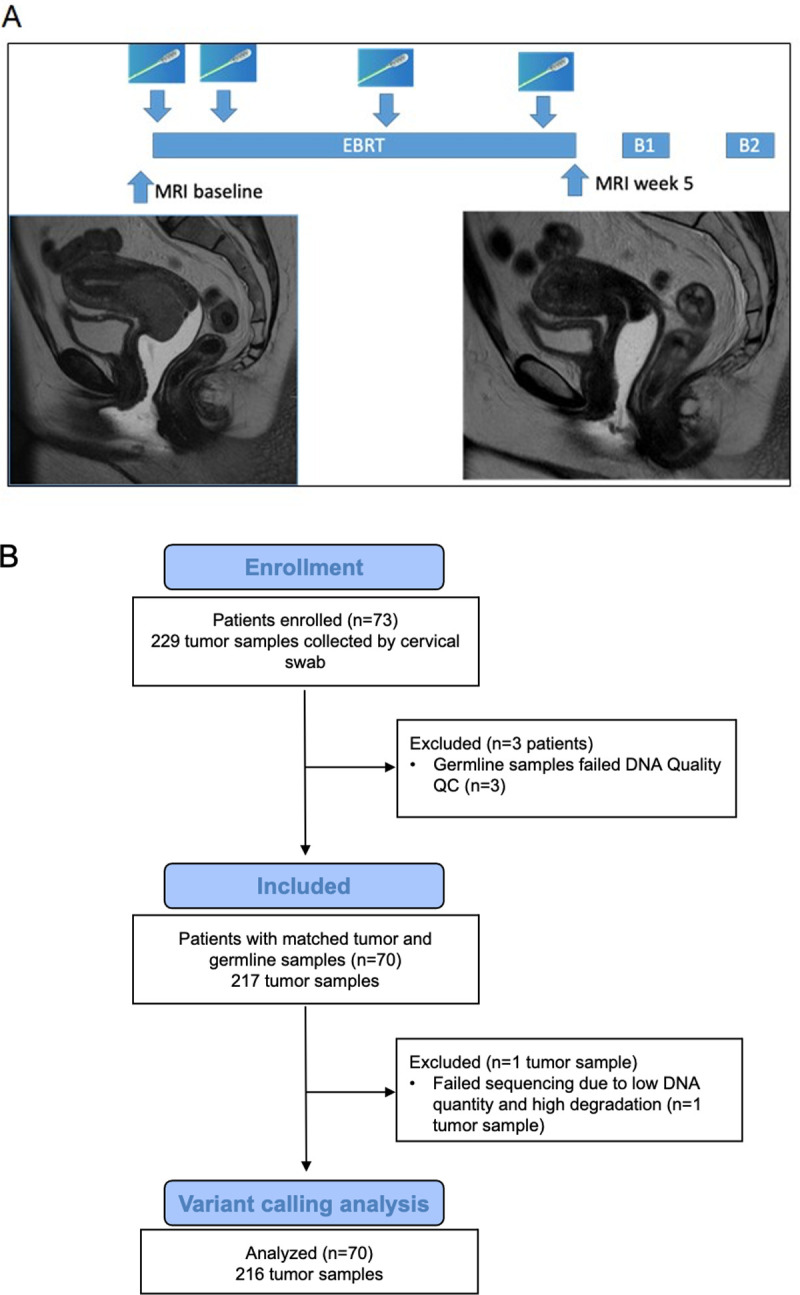
Overall study design and CONSORT diagram. (A) Patients with cervical cancer underwent five weeks of external beam radiation therapy (EBRT) followed by two brachytherapy treatments (B1 and B2), with swab samples collected at baseline, week 1, week 3, and week 5 of radiation therapy as well as week 12 (after completion of radiation therapy). (B) Of the 73 patients accrued on protocol, 70 patients with 217 total samples had DNA of adequate quantity and quality for sequencing. One tumor sample failed sequencing due to low quantity and high degradation and was not included in the analysis.

Patients were enrolled in an IRB-approved (2014–0543) multi-institutional prospective clinical trial at the University of Texas MD Anderson Cancer Center and the Harris Health System, Lyndon B. Johnson General Hospital Oncology Clinic from 2014–2019 (**Fig 1A, Table 1**). Inclusion criteria were newly diagnosed cervical cancer per the Federation of Gynecology and Obstetrics (FIGO) 2009 staging system, clinical stage IB1-IVA cancers, and visible, an exophytic tumor on speculum examination with planned definitive treatment of intact cervical cancer with external beam radiation therapy, cisplatin, and brachytherapy. Patients with any previous pelvic radiation therapy were excluded.

**Table 1:** Patient characteristics.

Characteristic	No. of patients	%
**Age at diagnosis**		
Median	47 years	
Range	28–91 years	
**Race/Ethnicity**		
African American	5	7%
American Indian/Alaskan Native	2	3%
Asian	2	3%
Hispanic/Latino	31	44%
White/Caucasian	30	43%
**Histology**		
Serous	1	1%
Adenocarcinoma	9	13%
Squamous cell carcinoma	60	86%
**2009 FIGO stage**		
IA1	1	2%
IB1	4	6%
IB2	12	17%
IIA	5	7%
IIB	31	44%
IIIB	12	17%
IVA	5	7%
**Tumor grade**		
I	3	4%
II	24	34%
III	24	34%
Unknown	19	27%
**Highest clinically involved nodal level on PET or CT**		
Para-aortic	4	6%
Common iliac	12	17%
External iliac	23	33%
Internal iliac	6	9%
Node-negative	22	31%
Unknown	3	4%
**Max tumor dimension on MRI, cm**		
Median	5	
Range	1.2–11.5	
Unknown	2	

Patients underwent standard-of-care pretreatment evaluation for disease staging, including tumor biopsy to confirm the diagnosis; pelvic magnetic resonance imaging (MRI) and positron emission tomography/computed tomography (PET/CT); and standard laboratory evaluations, including a complete blood cell count, measurement of electrolytes, and evaluation of renal and liver function. Patients received pelvic radiation therapy to a total dose of 40‒45 Gy delivered in daily fractions of 1.8 to 2 Gy over 4 to 5 weeks. Thereafter, patients received intracavitary brachytherapy with pulsed-dose-rate or high-dose-rate treatments. According to standard institutional protocol, patients received cisplatin (40 mg/m^2^ weekly) during external beam radiation therapy. Patients underwent repeat MRI at the completion of external beam radiation therapy or at the time of brachytherapy, as indicated by the extent of disease.

### Sample collection and DNA extraction

Isohelix swabs (product # DSK-50 and XME-50, www.isohelix.com, UKSamples) were brushed against the viable cervical tumor several times by a clinician from the department of radiation oncology or gynecologic oncology at either the University of Texas MD Anderson Cancer Center or Lyndon B. Johnson General Hospital Oncology Clinic. The isohelix swab has a unique matrix design that yields one to five micrograms of high-quality DNA sufficient for sequencing applications from a single swab of the tumor surface [14]. Patients underwent swabbing at baseline, the end of week 1 (after five fractions), at the end of week 3 (after 10–15 fractions), and within a week before the first brachytherapy treatment or at the time of brachytherapy (week 5), for a total of four swabs during radiation therapy (**Fig 1A**). Additionally, 9 patients had swabs collected at the first follow up visit after treatment completion (week 12). In each swabbing session, attention was taken to obtain samples from the same general tumor region. Normal buccal samples were collected once at baseline to identify germline mutations present in individual patients. DNA was extracted from normal buccal and cervical cancer samples per Isohelix # DSK-50 manufacturer’s instructions.

### Whole-exome sequencing and mutational analysis

Illumina WES sequencing was performed on normal buccal control and cervical tumor DNA swab samples. Captured libraries were sequenced on a HiSeq 4000 series (Illumina Inc., San Diego, CA, USA) on a TruSeq version 3 Paired-end Flowcell according to manufacturer’s instructions at a cluster density between 700–1000K clusters/mm^2^. Sequencing was performed for 2 × 100 paired-end reads with a 7-nucleotide read for indexes using Cycle Sequencing version 3 reagents (Illumina). The average coverage achieved with the Roche Nimblegen probes was 180 reads (range 50–359) for cervical tumor samples.

Paired-end raw sequence reads in fastq format were aligned to the reference genome (human Hg19) using Burrows-Wheeler Aligner (BWA) [15] with three mismatches with 2 in the first 40 seed regions for sequences less than 100 bp or using BWA-MEM with 31 bp seed length for sequences over 100 bp. The aligned BAM files were subjected to mark duplication, re-alignment, and re-calibration using Picard and the Genome Analysis Toolkit [16] before any downstream analyses.

**Fig 2:**
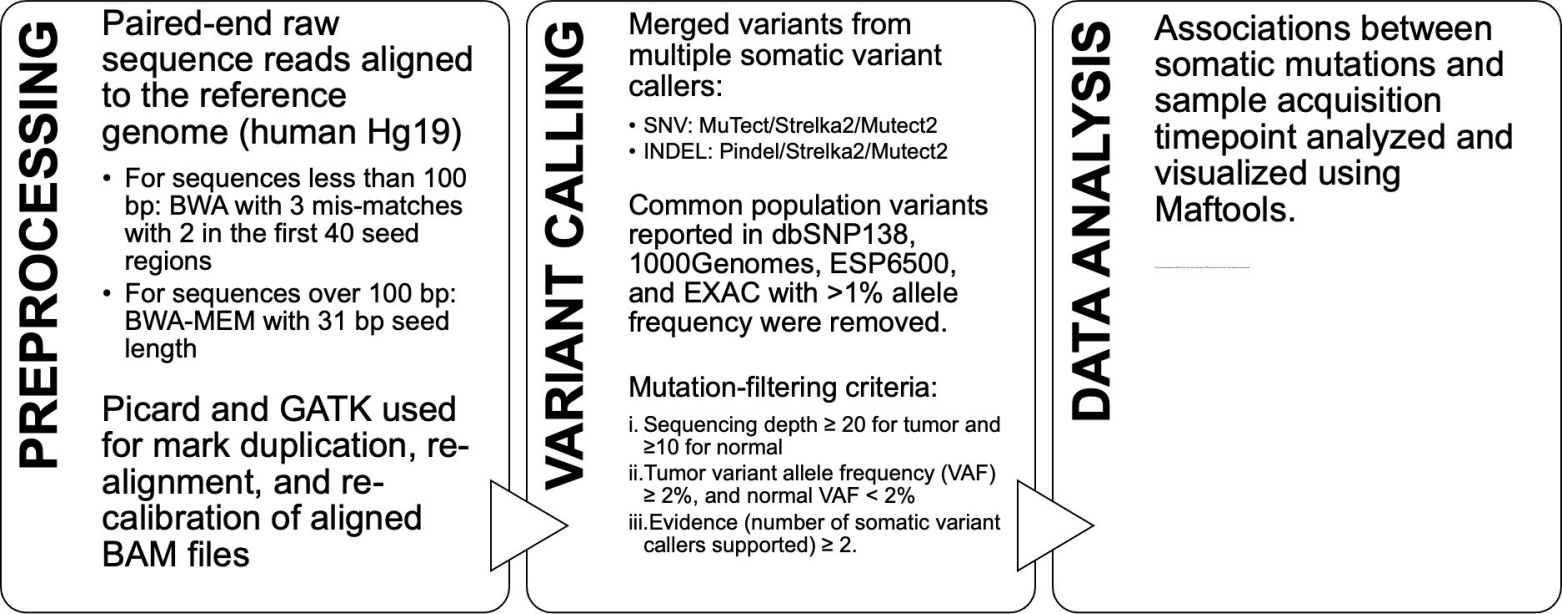
Computational pipeline for whole exome sequencing data. Workflow depicting preprocessing, variant calling and data analysis tools and parameters implemented to analyze WES data acquired from tumor DNA collected by cervical swab.

A custom computational pipeline was optimized for mutation calling (**Fig 2**). Based on the alignment results (BAM files) above, somatic mutations, including single-nucleotide variants (SNVs) and small insertions and deletions (INDELs), were obtained through merging variants from multiple somatic variant callers–MuTect, Pindel, GATK4 Mutect2, and Strelka2 [17–20]. Common population variants reported in dbSNP138, 1000Genomes, ESP6500, and EXAC with >1% allele frequency were removed. The following mutation-filtering criteria were applied for calling somatic mutations: (i) sequencing depth ≥ 20 for tumor and ≥10 for normal, (ii) tumor variant allele frequency (VAF) ≥ 2%, and normal VAF < 2%, (iii) Evidence (number of somatic variant callers supported) ≥ 2. Associations between somatic mutations and sample acquisition timepoint were analyzed and visualized using Maftools [21]. Tumor purity (TP), that is, the proportion of cancer cells in a tumor sample, was calculated from SNVs by the Tumor Purity Estimation (TPES) as well as from copy number profiles using Sequenza [22, 23] (**S1 Table**).

### Feasibility and statistical analysis

Feasibility was defined as greater than or equal to 85% of swab acquired tumor DNA samples successfully sequenced (objective 1) and successful processing through the above-described customized pipeline for inclusion in WES data analysis with at least 5% TP (objective 2). As this was an exploratory trial and the endpoints were primarily descriptive, no formal power analysis was performed. When comparing values between two timepoints, p-values were calculated by two-sided unpaired t-test. For comparison of quantitative changes at multiple time points, ANOVA testing was used to determine p-values.

## Results

### Patient and tumor characteristics

Clinicopathologic data are summarized in **Table 1**. Overall, 48 patients (68%) had advanced disease (stage IIB or greater), and most had squamous cell carcinoma with tumor grade II or higher. The median tumor size (based on the short axis diameter on pretreatment MRI) was 5cm (range 1.2‒11.5 cm). Forty-five patients had positive pelvic or para-aortic lymph nodes on PET or CT.

### DNA quality and sequencing characteristics of collected samples

**Fig 3:**
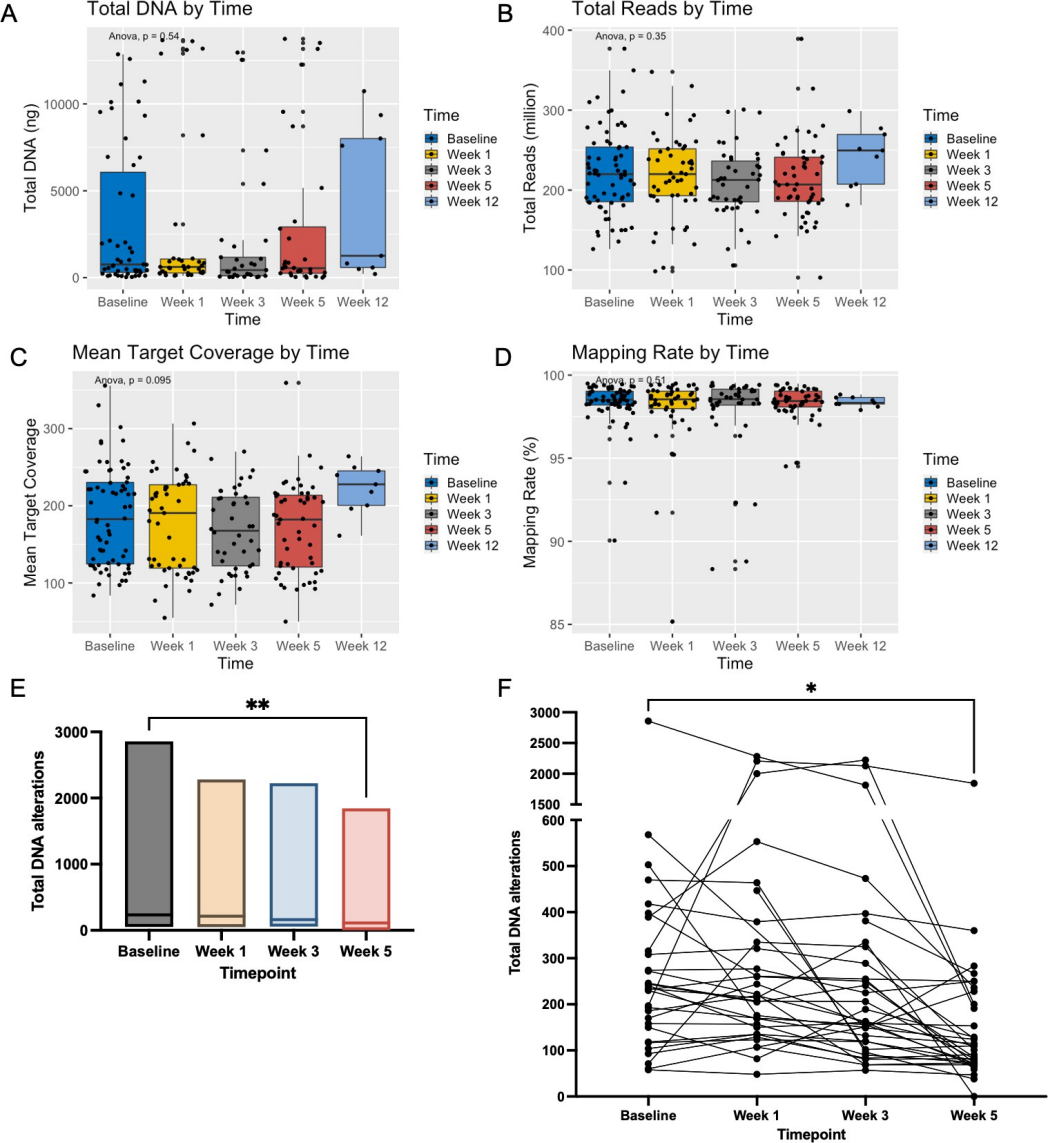
Quality Characteristics and DNA alterations for all Samples by Time (A) Total DNA yield did not differ by acquisition timepoint. Total reads (B), mean target coverage (C) and mapping rate (D) did not differ by timepoint. Number of DNA alterations at baseline decreased at week 5 of CRT in all patients (E, p = 0.008) as well as when 33 patients with samples at all 4 time points were examined (F, p = 0.03).

Two hundred twenty-nine total tumor samples were collected (**Fig 1**). For 3 patients, matching normal (buccal) samples failed either DNA quality or quantity quality check (QC), and thus 12 paired tumor samples were excluded. Two hundred seventeen total tumor samples and 70 normal germline samples were sequenced. Of these, 161 (74%) had both optimal DNA quality and quantity QC, 54 (25%) had suboptimal quantity, but adequate quality and 2 (1%) had optimal quantity but low-quality DNA. 1 sample failed sequencing due to both suboptimal DNA quality and quantity; thus, 216 total tumor samples (94%) from 70 patients were included in the analysis (**Fig 1B**). Median DNA concentration per sample was 12 ng/uL (range 1.5–167 ng/uL). The mean total reads per sample was 216 million (+/-50), and mean mapping rate was 98.15% (+/-1.84) (**Table 2**). Total DNA yield was not different by timepoint (p = 0.54), nor were total reads (p = 0.35), mapping rate (p = 0.51), or mean target coverage (p = 0.10) (**Fig 3A–3D**). No significant differences were detected by sequencing batch.

**Table 2:** Quality characteristics for samples by timepoint.

	Baseline	Week 1	Week 3	Week 5	Week 12
	(N = 66)	(N = 49)	(N = 42)	(N = 51)	(N = 9)
**DNA concentration (ng/uL)**					
Mean/SD	39.42 ± 49.73	29.98 ± 46.48	22.86 ± 35.10	28.70 ± 45.12	56.34 ± 48.28
Median	14.5	13.4	8.65	9.1	27.5
**Total DNA yield (ug)**					
Mean/SD	3.15 ± 4.13	2.64 ± 4.63	1.82 ± 3.44	2.90 ± 4.56	4.34 ± 4.44
Median	0.76	0.62	0.44	0.55	1.26
Unknown	16 (24.24%)	14 (28.57%)	13 (30.95%)	19 (37.25%)	0 (0.00%)
**Total Reads (million)**					
Mean/SD	222.88 ± 53.26	218.06 ± 52.06	209.83 ± 43.11	212.85 ± 49.68	242.42 ± 38.15
Median	219.95	219.95	212.68	206.95	249.53
**Mapping Rate (%)**					
Mean/SD	98.33 ± 1.37	97.99 ± 2.31	97.81 ± 2.59	98.36 ± 0.96	98.39 ± 0.30
Median	98.515	98.54	98.55	98.44	98.33
**Mean Target Coverage (per sample)**					
Mean/SD	186.60 ± 64.26	176.10 ± 62.73	168.56 ± 52.51	171.78 ± 58.96	222.53 ± 32.11
Median	182.7	190.62	167.655	182.17	227.84
**Insertions/ Deletions (Count)**					
Mean/SD	41.00 ± 20.07	41.61 ± 30.22	39.07 ± 28.99	32.62 ± 14.32	55.00 ± 38.11
Median	37	36	34.5	30.5	43
**Substitutions (Count)**					
Mean/SD	301.14 ± 400.86	353.02 ± 523.01	283.83 ± 503.63	140.92 ± 259.59	54.56 ± 39.75
Median	177	165	138.5	66	45
**TPES Tumor Purity**					
Mean/SD	0.62 ± 0.21	0.60 ± 0.19	0.61 ± 0.25	0.54 ± 0.23	0.69 ± 0.19
Median	0.635	0.59	0.615	0.56	0.72
**Sequenza Tumor Purity**					
Mean/SD	0.28 ± 0.20	0.27 ± 0.20	0.23 ± 0.17	0.23 ± 0.16	0.19 ± 0.11
Median	0.195	0.18	0.15	0.19	0.12

### Mutation characteristics for all samples

Sixty-six patients had samples analyzed at baseline, 49 at Week 1, 42 at Week 3, 51 at Week 5, and 9 at Week 12. 33 patients had samples analyzed at all four time points. Mean number of substitutions, insertions and deletions over time was 342 (range 50–2,857) at baseline, 394 (range 48–2,281) at Week 1, 232 (range 57–2,223) at Week 3, 170 (range 0–1,843) at Week 5, and 110 (range 53–300) at Week 12 (**Table 2, S1 Table**). While total DNA alterations numerically decreased at Week 1 and Week 3 compared to baseline, this was not statistically significant (p = 0.54 and 0.86 respectively). There was a statistically significant decrease in total DNA alterations at baseline to Week 5 in all patients (p = 0.008) as well as patients with all 4 timepoints through the course of CRT (p = 0.03, **Fig 3E and 3F**).

Median TP of all samples as calculated by the Sequenza algorithm at baseline was 0.195 (range 0.10–0.99) and 0.190 (range 0.10–0.98) at week 5 (p = 0.07; **Table 2, S1 Table**). Median TP of all samples as calculated by the TPES algorithm at baseline was 0.62 (range 0.17–0.99) and 0.54 (range 0.12–0.97) at week 5 (p = 0.07; **Table 2, S1 Table**).

**Fig 4:**
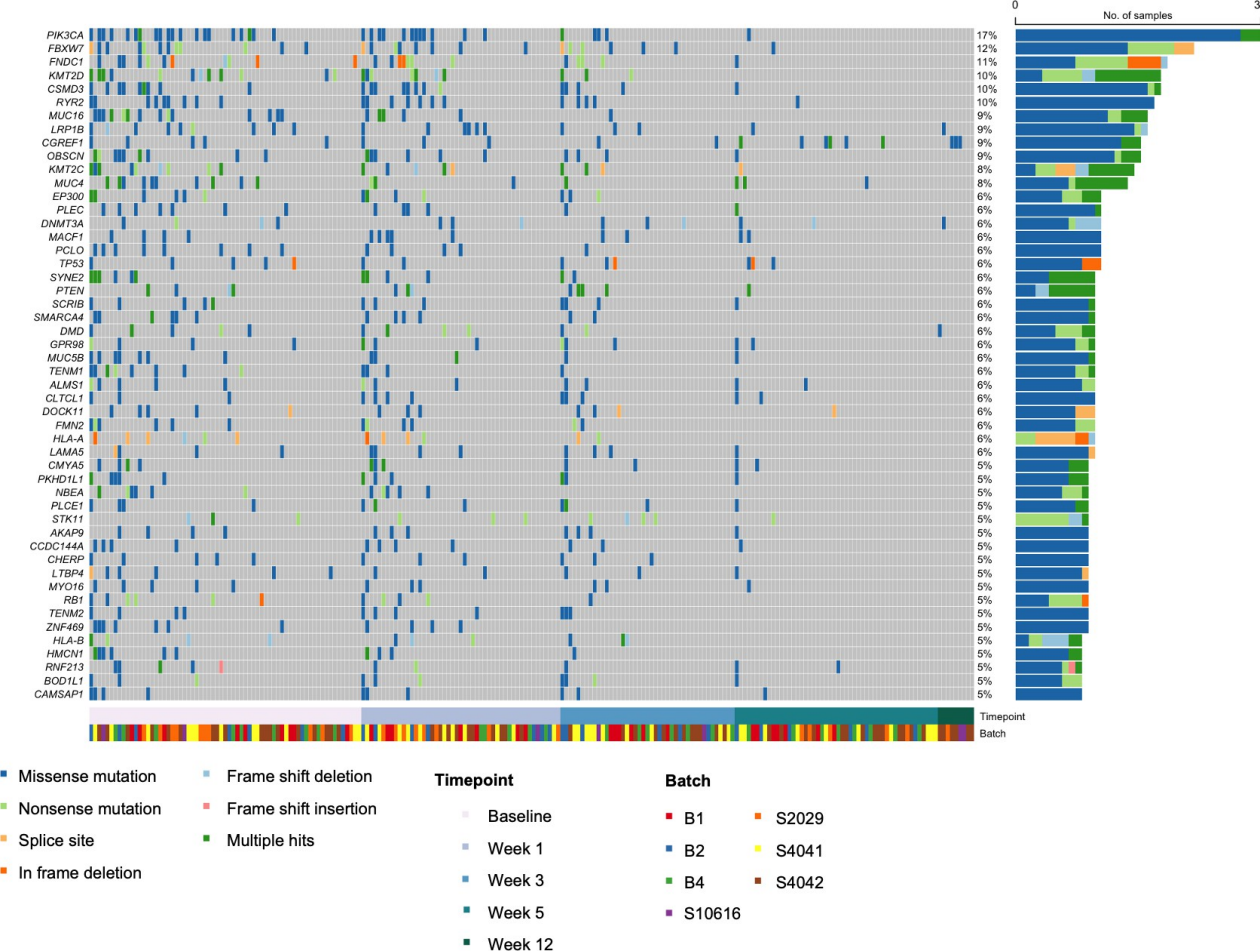
Top 50 gene alterations over time for 70 patients with paired normals. Heat map displaying the top 50 genes ranked by occurrence for 70 patients (216 samples) grouped by timepoint collection during chemoradiation.

**Fig 5:**
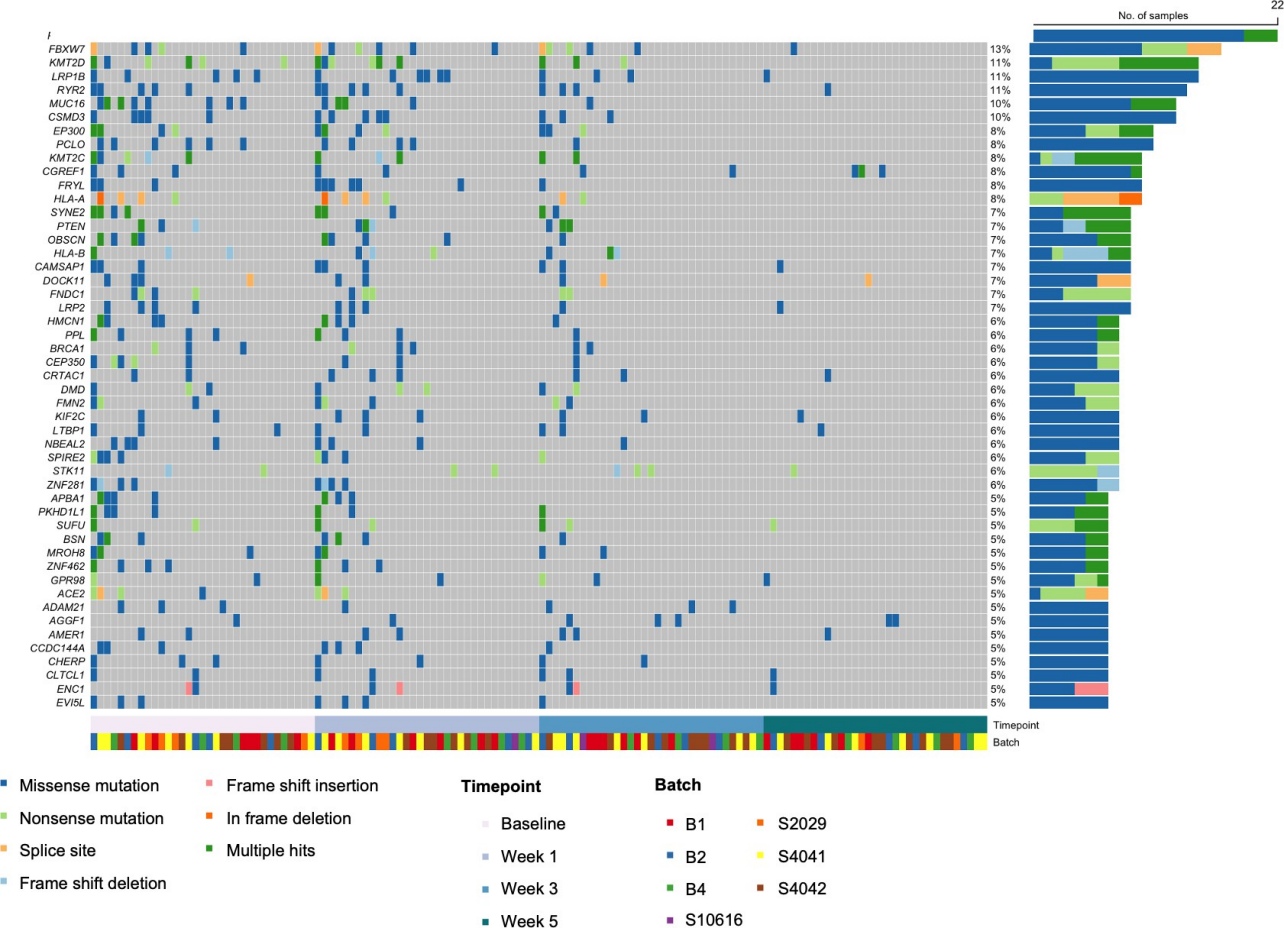
Top 50 gene alterations over time for 33 patients with all four-time points. Heat map displaying the top 50 genes ranked by occurrence for 33 patients (132 samples) grouped by timepoint collection during chemoradiation.

Across analyzed samples (n = 216), the top 5 most common alterations were in PIK3CA, FBXW7, FNDC1, KMT2D, and CSMD3 (**Fig 4, S2 Table**). For 33 patients with samples available at all 4 time points (n = 132 samples), the top 10 most common alterations were in PIK3CA (17%), FBXW7 (13%), KMT2D (11%), LRP1B (11%), RYR2 (11%), MUC16 (10%), CSMD3 (8%), EP300 (8%), PCLO (8%), and KMT2C (8%) (**Fig 5**). Of these, the remaining detectable alterations at week 5 were in FBXW7, LRP1B, and RYR2. From the top 50 alterations, other genes with alterations remaining at week 5 included CGREF1, CAMSAP1, DOCK11, LRP2, CRTAC1, KIF2, LTPB1, STK11, SUFU, GPR98, AGGF1, AMER1, CLTCL1, and ENC1.

## Discussion

To our knowledge, this is the first study describing the use of non-invasive swab-based cervical tumor sample collection and performance of serial WES over the course of CRT. TP analysis confirmed the presence of tumor cells captured and sequenced at timepoints throughout the course of treatment, and gene alterations present at baseline and persistent through the course of treatment were identified. We anticipate that this methodology will allow for comprehensive characterization of changes in the mutational landscape of cervical cancers in patients undergoing treatment.

A limitation of the current study is the absence of tumor biopsy samples with which to compare DNA yield, quality, and sequencing output in swab-acquired samples. A previously reported study comparing various tumor sample acquisition methodologies has shown that DNA yield and quality recovered from isohelix swabs is comparable to alternative techniques including other commercially available brushes (Rover brush [24]), formalin-fixed, paraffin-embedded (FFPE) tissue, and flash frozen tissue biopsy [25]. Mean DNA yield from samples collected by isohelix swabs of the oral cavity was 0.76ug, while mean DNA yield from isohelix swabs of the cervix reported here was 2.8ug when evaluating 5 sampling time points. Likewise, in a comparison of DNA quality, DNA recovered from isohelix swabs and FFPE had comparable mapping rate (67% for FFPE samples and 71% for samples acquired by isohelix swab) [25]. In our study, the mean mapping rate was 98.2% (range 85.2–99.5%) among all time points. In both the previous study and the current one, swab-based sampling provided adequate DNA yield for successful WES. Variations in quantitative DNA yield and mapping rates between the current study and others may be due to the variation in eluting volumes, DNA extraction and preparation kits, and sequencing techniques.

An additional study comparing sample acquisition by biopsy and isohelix brush swab for cancers in the oral cavity for epigenome-wide DNA methylation found no significant difference in DNA yield between tissue and brush samples and matched tissue. Isohelix brush swabs had an excellent correlation in the oral cavity [10]. Mean DNA yield from frozen tissue was 0.39ug (range 0.19–0.66ug), and 0.53ug (range 0.51–2.00ug) from swab-acquired DNA. Genomic DNA from both swabs and frozen tissues had similar mapping efficiency with over 90% mapping efficiency for swab-acquired DNA. Investigators successfully identified potential prognostic and predictive biomarkers for malignant lesions in the oral cavity with high sensitivity and specificity using a comparable isohelix swab design as was used here [10]. While isohelix brushes have been designed and marketed for DNA acquisition of the buccal mucosa, we were able to acquire sufficient quality and quantity DNA for WES in all but one tumor sample with collected samples by isohelix brushings from cervical mucosa. This method could rapidly be applied to the study of other gynecologic, head and neck, anorectal, and skin malignancies.

Past studies that relied on serial biopsies have been limited by logistical challenges, concerns about patient discomfort, and complications from traditional biopsies, including bleeding and infection. In a report by Weidhaas et al., gene expression profiling was performed on tissue biopsies collected from 13 patients pre- and mid-treatment and investigators identified a seven gene signature that predicted improved local control [26]. In a similar study of patients with locally advanced cervical cancer undergoing CRT, investigators acquired biopsies pretreatment and at week 3 of CRT and performed RNA-sequencing on 20 matched pairs. They found that patients who succumbed to disease at the time of their report had enrichment of gene expression from mitotic pathways and increased retention of HPV E6/E7 gene expression at week 3 of CRT, which may promote treatment resistance [27]. No analysis of somatic mutations was performed in either of these studies. Our novel, non-invasive technique allows DNA to be collected from cervical swabs, reducing obstacles to serial biopsy collections. Furthermore, while previous studies have recognized gene expression signatures associated with long-term patient outcomes, this is the first study to report findings from WES of cervical tumor samples collected longitudinally over the course of CRT.

Encouragingly, when mutation profile of swab acquired tumor samples from 23 patients in our preliminarily analyzed cohort was compared with those obtained from cervical cancer patients in The Cancer Genome Atlas (TCGA) as a comparison group, we found that more than 93% of mutated genes detected at baseline were also present in the TCGA group (**S1 Fig**). Likewise, several canonical genes known to promote cervical cancer pathogenesis including Rb and p53 were detected in our dataset [28]. Phosphoinositide 3-kinase (PI3K) pathway-related mutations were also prevalent (**S2 Fig**). Complementing our findings, previous studies have reported perturbations in *PIK3CA*, an oncogene part of the ERBB2/PI3K/AKT/mTOR pathway with a battery of druggable targets, present in over 50% of cervical tumor and cervical cancer cell lines [3].

More interestingly, serial tumor profiling also allowed us to identify a panel of mutated genes enriched and present at baseline and persisting through the end of CRT including FBXW7, LRP1B, and RYR2. Mutations in FBXW7 which encodes a protein involved in ubiquination and proteasome degradation of oncoproteins as well as DNA double strand break response [29], are frequently present in cervical cancers and loss of function has been correlated with chemotherapy resistance and poor clinical outcomes [30, 31]. Likewise, LRP1B mutations have frequently been identified in cancers including cervical cancer and while high mutation rates have been found to correlate with worse prognosis in hepatocellular carcinoma and glioblastoma [32] they have also been associated with improved patient response to immunotherapies in multiple cancer types including prostate, melanoma, and non-small cell lung cancer [33, 34]. The role of RYR2 in cervical cancer has not yet been described but has previously been identified as a frequently altered gene in cervical cancer samples collected for the TCGA database [35]. Time dependent changes in these mutational signatures would have been impossible to detected in pretreatment biopsies alone and may open doors to future therapeutic interventions.

As expected, TP estimates for swab acquired samples were lower, however, comparable to previously reported estimates from biopsy samples from the TCGA analysis in biopsy samples [36] and decreased over the course of therapy. Even in the case of biopsies, often considered the "gold-standard" method for cancer cell sampling, TP values can display wide range and variance. For example, biopsy-sourced cells from both cervical cancer and other solid tumors that underwent WES demonstrated TP values ranging from 0.21 to 1.0 [37, 38].

While several computational methods exist to infer TP, these methods differ in the types of genomic information used, such as gene expression, SCNA, and somatic mutations [39, 40]. To reduce the systematic bias of genomic investigation of samples containing both tumor and non-neoplastic tissue, TP levels are often considered during analysis to deconvolute contaminant contributions from the tumor microenvironment field [41–43]. Thus, we evaluated two purity estimation tools with differing prediction mechanisms to better understand the cellular heterogeneity within our swab acquired samples. The Sequenza tool was developed for both exome and whole-genome deep sequencing of tumor DNA where average depth ratio in tumor versus normal samples and allele frequency is used to estimate for cellularity and ploidy [23]. Investigators found that Sequenza correctly detected ploidy in samples with as low as 30% tumor content. TPES predicts TP from variant allelic fraction distribution of SNVs to more accurately predict TP when tumor genomes are copy-number neutral or euploid [22]. This tool was validated on WES data from TCGA tumor samples and enabled TP estimation in samples that failed TP prediction algorithms dependent on somatic copy-number alterations. TPES estimates were enriched in samples with low genomic burden, while SCNA-based tools similar to Sequenza were more proficient with high genomic burden cases suggesting a complementary role for these two tools in the analysis of tumor WES data. Indeed, previous work investigating differences in TP algorithms have supported the use of a combined value when multiple estimations are performed [36]. Moving forward, when applying TP estimations to describe dynamic mutational changes over time, we propose to utilize Sequenza estimations given the low tumor content of our samples and expected high genomic mutation burden in cervical cancer samples, and reserve TPES or other alternative algorithms for samples that fail the Sequenza prediction algorithm.

There are several limitations to this study that must be addressed. First, tumor samples were not available at all four time points for 37 patients due to the inability to procure samples. Second, long-term follow-up data is not available for this cohort at this time. Finally, while the clinical significance of individual mutations was not the focus of the present study, studies are underway to identify signatures and mutations associated with radiation sensitivity and resistance among women with differential responses to CRT for cervical cancer. For this future work, we hypothesize that mutations that survive the initial weeks of radiation treatment may be clinically relevant drivers of radiation resistance, and we plan to focus on characterizing the clonal architecture of residual tumors to identify more granular molecular signatures predictive of treatment response. This will permit future investigations of treatment escalation or de-escalation in appropriate populations.

## Conclusion

In conclusion, this work provides proof of concept that a noninvasive, swab-based biopsy technique can be utilized to serially sample tumors for in-depth sequencing analysis. This novel methodology can be added to the translational research armamentarium to interrogate tumor genetics and eventually tailor cancer-directed therapies.

## Supporting information

S1 Checklist CONSORT 2010 checklist of information to include when reporting a pilot or feasibility trial*. (PDF)

S1 Table Sample and sequencing quality control metrics and tumor purity estimates for each tumor sample. (XLSX)

S2 Table Gene list with alteration type and frequency for all samples. (CSV)

S1 Fig Overall gene alterations from swab acquired tumor samples (patients 2–30) is similar to landscape of TCGA cervical squamous cell carcinoma dataset. (A) Ninety-four percent (1339/1430) of altered genes in baseline samples (defined as substitutions, insertions or deletions in gene) were also identified in the TCGA dataset, suggesting accurate identification of mutated genes related to cervical cancer. (B) The distribution of the top 30 most altered genes in study samples 2–30 and in TCGA(C) was also similar.
(TIF)

S2 Fig Lollipop plots showing mutation site, type, and frequency of canonical genes associated with cervical cancer pathogenesis for all samples. (TIF)
