# Supplementary figures and images for: Feasibility of a novel non-invasive swab technique for serial whole-exome sequencing of cervical tumors during chemoradiation therapy
Source: PLoS One. 2022 Oct 6;17(10):e0274457. doi: 10.1371/journal.pone.0274457 (PMC9536567; doi:10.1371/journal.pone.0274457)

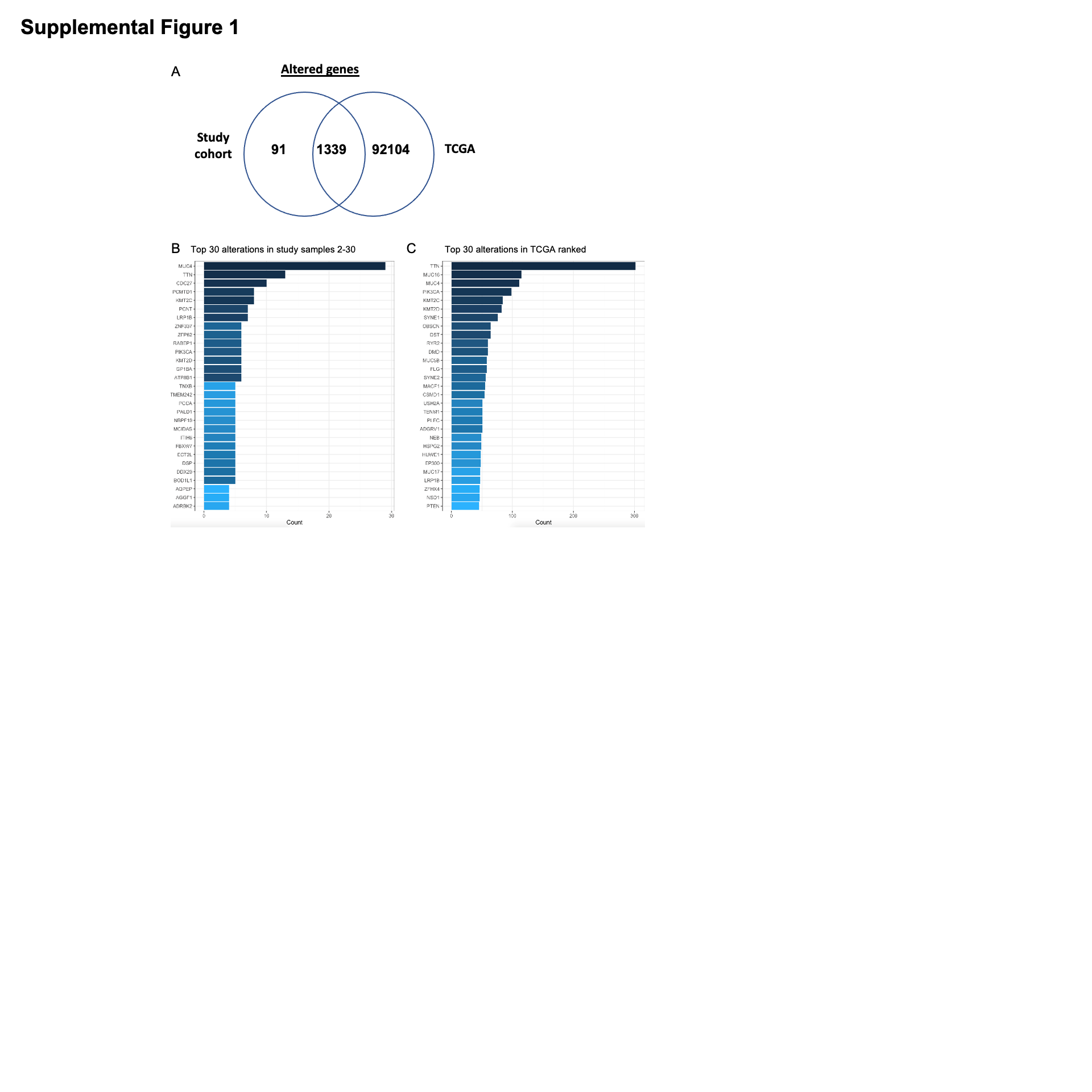

Supplement: S1 Fig — (A) Ninety-four percent (1339/1430) of altered genes in baseline samples (defined as substitutions, insertions or deletions in gene) were also identified in the TCGA dataset, suggesting accurate identification of mutated genes related to cervical cancer. (B) The distribution of the top 30 most altered genes in study samples 2–30 and in TCGA(C) was also similar. (TIF) [file pone.0274457.s004.tif]

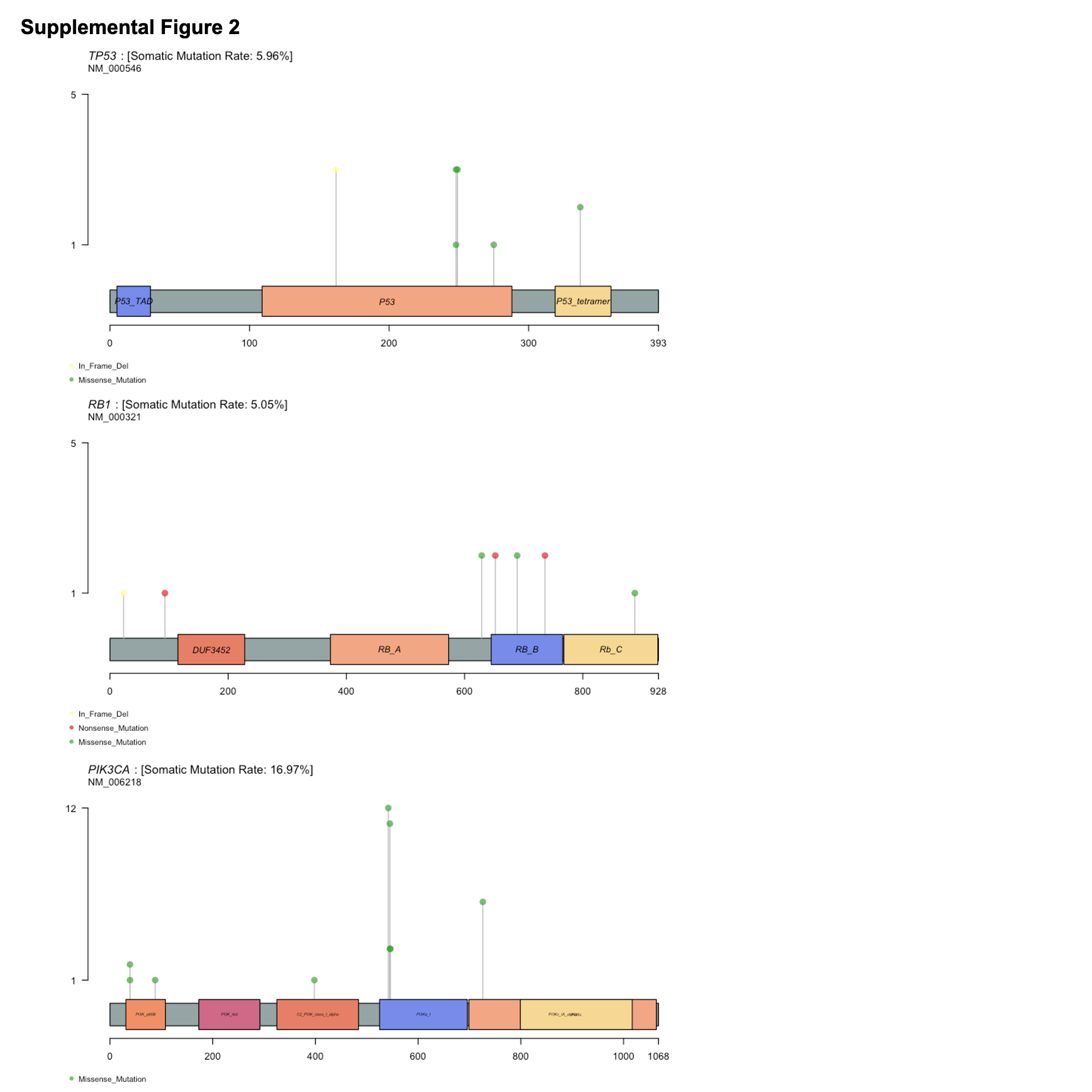

Supplement: S2 Fig — (TIF) [file pone.0274457.s005.tif]
